# Sildenafil Inhibits the Growth and Epithelial-to-mesenchymal Transition of Cervical Cancer *via* the TGF-β1/Smad2/3 Pathway

**DOI:** 10.2174/1568009622666220816114543

**Published:** 2023-01-01

**Authors:** Ping Liu, Jing-Jing Pei, Li Li, Jing-Wei Li, Xiao-Ping Ke

**Affiliations:** 1 Department of Obstetrics and Gynecology, Yangpu Hospital, School of Medicine, Tongji University, Shanghai, 200090, China;; 2 Child Healthcare Department, The Affiliated Wuxi Matemity and Child Health Care Hospital of Nanjing Medical University, Wuxi 214001, China

**Keywords:** Sildenafil, cervical cancer, epithelial-to-mesenchymal transition, invasion, migration, platinum

## Abstract

**Aims:**

The study aims to explore new potential treatments for cervical cancer.

**Background:**

Cervical cancer is the second most common cancer in women, causing >250,000 deaths worldwide. Patients with cervical cancer are mainly treated with platinum compounds, which often cause severe toxic reactions. Furthermore, the long-term use of platinum compounds can reduce the sensitivity of cancer cells to chemotherapy and increase the drug resistance of cervical cancer. Therefore, exploring new treatment options is meaningful for cervical cancer.

**Objective:**

The present study was to investigate the effect of sildenafil on the growth and epithelial-to-mesenchymal transition (EMT) of cervical cancer.

**Methods:**

HeLa and SiHa cells were treated with sildenafil for different durations. Cell viability, clonogenicity, wound healing, and Transwell assays were performed. The levels of transforming growth factor-β1 (TGF-β1), transforming growth factor-β type I receptor (TβRI), phosphorylated (p-) Smad2 and p-Smad3 in cervical cancer samples were measured. TGF-β1, Smad2 or Smad3 were overexpressed in HeLa cells, and we measured the expression of EMT marker proteins and the changes in cell viability, colony formation, *etc*. Finally, HeLa cells were used to establish a nude mouse xenograft model with sildenafil treatment. The survival rate of mice and the tumor size were recorded.

**Results:**

High concentrations of sildenafil (1.0-2.0 μM) reduced cell viability, the number of HeLa and SiHa colonies, and the invasion/migration ability of HeLa and SiHa cells in a dose- and time-dependent manner. The expression of TGF-β1, TβRI, p-Smad2 and p-Smad3 was significantly enhanced in cervical cancer samples and cervical cancer cell lines. Sildenafil inhibited the expression of TGF-β1-induced EMT marker proteins (Snail, vimentin, Twist, E-cadherin and N-cadherin) and p-Smad2/3 in HeLa cells. Overexpression of TGF-β1, Smad2, and Smad3 reversed the effect of sildenafil on EMT, viability, colony formation, migration, and invasion ability of HeLa cells. In the *in vivo* study, sildenafil significantly increased mouse survival rates and suppressed xenograft growth.

**Conclusion:**

Sildenafil inhibits the proliferation, invasion ability, and EMT of human cervical cancer cells by regulating the TGF-β1/Smad2/3 pathway.

## 1 INTRODUCTION

Cervical cancer is the second most common cancer in women, with an estimated 570, 000 cases of cervical cancer and 311, 000 deaths from the disease occurring in 2018, making it the fourth most common cancer in women [1]. In 2020, WHO released the global strategy to accelerate the elimination of cervical cancer as a public health problem, and 194 countries promised together to eliminate cervical cancer [2]. Cervical cancer includes squamous cell carcino- ma (70%), cervical adenocarcinoma (25%) or mixed histological tumors (5%) [3]. It was reported that cervical cancer took the lives of 4,138 women in the United States in 2018 [4]. The disease is even more severe in developing countries in terms of higher incidence. In China, there are ~140,000 new cases of cervical cancer each year and ~37,000 recorded deaths [5]. Although human cervical cancer vaccines (*e.g.,* Gardasil and Cervarix) can prevent human papillomavirus (HPV) infection, they have no therapeutic effect on patients who have been infected with HPV and patients with precancerous lesions, nor can they prevent all subtypes of HPV infection [6]. At present, patients with cervical cancer are mainly treated with platinum compounds [7], which often cause severe adverse reactions related to neuro-, nephritic, hematic, and immune toxicity [8]. Furthermore, the long-term use of platinum compounds can reduce the sensitivity of cancer cells to chemotherapy and increase the drug resistance of cervical cancer [9]. Therefore, it is crucial to further elucidate the molecular mechanism underlying cervical cancer occurrence and development and identify new treatment options.

Phosphodiesterase 5 (PDE5) is highly expressed in a variety of malignant tumors, such as bladder cancer, breast cancer, non-small cell lung cancer, and colorectal cancer, indicating that PDE5 plays an important role in tumorigenesis and that inhibition of PDE5 activity may have antitumor effects [10, 11]. Studies have shown that sildenafil, an inhibitor of PDE5, induces apoptosis of some cancer cells through nitric oxide (NO)/PDE5-dependent manner [12]. Transforming growth factor (TGF)-β1 is a multifunctional cytokine that plays a dual role in cell proliferation and differentiation regulation [13]. TGF-β1 is involved in angiogenesis, immune suppression, damage repair and extracellular matrix formation, fibrosis and tumorigenesis [14]. Some scholars have found that the majority of cancer patients exhibit high expression of TGF-β1 in the tumors and plasma. Breast cancer cells show elevated expression of TGF-β1, which augmented the expression of EMT markers in CAFs with the reduction in E-cadherin [15]. TGF-β1 was also found to be overexpressed in pancreatic cancer [16] and highly expressed in cultured lung, colon, liver, and kidney cancer tissues or cells. Hazelbags *et al.* found that pathological sections of cervical cancer tissues were positive for TGF-β1 mRNA expression and suggested that, in addition to controlling cell proliferation, TGF-β1 is involved in the epithelial-to-mesenchymal transition (EMT) and migration of cervical cancer cells [17, 18]. Tjiong *et al.* found that the expression of TGF-β1 in cervical cancer tissue was significantly higher compared with that in the tissues of patients with chronic cervicitis and cervical intraepithelial neoplasia [19]. Another study found that the TGF-β1 treatment reduced the migration and invasion of cervical cancer cells [20]. These studies indicated that TGF-β1 plays an important role in the occurrence and development of cervical cancer.

As TGF-β1 plays an important role in the development of cervical cancer, the aforementioned results indicated that sildenafil may inhibit cervical cancer by regulating TGF-β1 expression. However, the effect and mechanism of action of sildenafil in the development of cervical cancer have not been investigated to date. Therefore, the present study was undertaken to investigate the effect of sildenafil on the proliferation, EMT, apoptosis, and cell cycle progression of cervical cancer cells. Its effect on the expression of EMT- and TGF-β1/Smad2/3 pathway-related proteins and the growth of cervical cancer xenograft was also examined to explore the underlying mechanism.

## 2 MATERIALS AND METHODS

### 2.1 Cell Treatment

Cervical cancer cells (HeLa, HT-3, C33A, SiHa and U14 cells) and human cervical epithelial cells (HCerEpiC) were purchased from The Cell Bank of Type Culture Collection of The Chinese Academy of Sciences (Shanghai, China). The cells were cultured in Dulbecco's modification of Eagle's medium (DMEM) containing 10% fetal bovine serum (FBS), 100 U/l penicillin, and 100 mg/l streptomycin and were cultured in a 5% CO_2_ cell incubator at 37 ^o^C. Sildenafil (Sigma-Aldrich; Merck KGaA, St. Louis, MO, USA) was diluted with dimethyl sulfoxide and added to the DMEM to obtain desired concentrations (0.5, 1.0, 1.5 and 2.0 μM).

### 2.2 Cell Viability Assay

The cell viability was measured with a method similar to Li *et al.* [21]. HeLa and SiHa cells in the logarithmic growth phase were digested, then centrifuged at 1,200 *×g* for 5 min at room temperature. The collected cell precipitates were re-suspended in DMEM containing 10% fetal bovine serum (Sigma-Aldrich; Merck KGaA, St. Louis, MO, USA) to make a single-cell suspension. The cell count was adjusted to 4×10^4^ cells/ml by a cell counting plate, and 100 μl cell suspension was added to each well. The 96-well plates were placed in a 5% CO_2_ incubator for 24 h. When the cells in the well became confluent, the old medium was discarded with a pipette gun. In each well, 100 μl of solution with different concentrations of sildenafil was added, and the control group was set with 0.01% dimethyl sulfoxide (DMSO). Subsequently, the 96-well plate was kept in the incubator for 12-72 h. The 96-well plates were taken out at the pre-set time, the original medium was discarded, and the 10 μl CCK-8 reagent (Sigma-Aldrich; Merck KGaA, St. Louis, MO, USA) was added to each well. The 96-well plates were put back in the cell incubator for incubation at 37 ^o^C for 2 h. Finally, the absorbance value (OD value) of HeLa and SiHa cells at 450 nm was measured on a microplate reader (BioTek, Winooski, Vermont, USA), and the cell viability of each group was calculated.

### 2.3 Clonogenicity Assay

The clonogenicity assay was performed with a method similar to Ashinuma *et al.* [22]. HeLa and SiHa cells in the logarithmic growth phase were digested, then centrifuged at 1,200 *×g* for 5 min at room temperature. The cell precipitates were collected and resuspended with a complete medium to make a single-cell suspension, and the cell number was adjusted to 1×10^4^/ml. Then, 1,000 cells per well were inoculated into 6-well plates, and 2 ml of DMEM with 10% fetal bovine serum was added to each well. The plates were gently shaken to disperse the cells evenly and were then placed into a 5% CO_2_ incubator for overnight culture at 37 ^o^C. After the cells were completely adherent to the plate, the old medium was discarded, and each well was treated with a 2 ml medium containing different concentrations of sildenafil. The 6-well plate was put back into the cell incubator, and cell proliferation was observed at intervals of 2-3 days for 12 days. Finally, the 6-well plate was taken out of the incubator, the old culture medium was discarded, and the plate was washed with 2 ml PBS three times. Following fixation with 4% paraformaldehyde for 30 min and washing with 2 ml PBS three times, crystal violet solution (0.5%) was added and the plate was left to stand in the dark for 15 min, followed by washing with 2 ml PBS for three times and drying. The colonies were counted using a stereomicroscope (ZOOM 2000, Leica Microsystems, Inc. Buffalo Grove, IL, USA).

### 2.4 Wound Healing and Transwell Assay

For the wound healing assay, similar to Meng *et al.* [23], HeLa and SiHa cells in the logarithmic growth phase were digested, centrifuged (1,200 *×g* for 5 min at room temperature), and resuspended in a complete medium. The cell suspensions were inoculated into 6-well plates. A total of 2 ml of DMEM with 10% fetal bovine serum was added to each well and cultured in 37 ^o^C, 5% CO_2_ incubator. The cells were observed under an Olympus BX50 microscope (Olympus, Tokyo, Japan, ×100) until a confluent monolayer was formed. Then, the cell monolayer was carefully scratched using a sterile 200-µl plastic pipette, and the cell fragments were removed by washing with 2 ml PBS twice. A total of 2 ml of previously prepared DMEM (without fetal bovine serum) containing different concentrations of sildenafil was added to each well to treat the cells. Three multiple wells were set for each concentration, and the 6-well plate was put back into the cell incubator for 24 h, after which it was observed under an Olympus BX50 microscope (Olympus, Tokyo, Japan, ×100) and photographed using Nikon Coolpix 990 camera (Nikon Corporation, Tokyo, Japan). The horizontal distance between the edges of the scratch was measured, and the migration rate of the cells was calculated.

For the Transwell assay, similar to Meng *et al.* [23], HeLa and SiHa cells were cultured with either medium alone (Control) or medium supplemented with sildenafil (0.5, 1.0, 1.5 or 2.0 μM) for 48 h, and then the cell invasion ability was measured using a Transwell chamber (Corning, Inc., Corning, NY, USA) assay. The upper chamber was coated with 100 µl diluted Matrigel (diluted with FBS-free MEM-a, 5:1) for 2 h at 37 ^o^C, whereas the lower chamber was filled with 500 µl of MEM-a supplemented with 20% FBS. Cells were then seeded to the upper chamber in FBS-free MEM-a and placed in an incubator at 37 ^o^C. The filter inserts were removed from the chambers 24 h later, and the cells remaining in the upper chamber were gently wiped off with a cotton swab. The cells in the bottom chamber were fixed with 4% paraformaldehyde and stained with crystal violet solution (0.5%). A total of six fields of view were randomly selected under an inverted microscope (magnification, ×100) for statistical analysis.

### 2.5 Patients and Specimen Collection

Samples were collected from 30 patients who were diagnosed with cervical cancer and treated at the Yangpu Hospital between January 2018 and January 2020. For each patient, control samples were obtained from adjacent non-cancerous tissue at a distance of at least 5 cm from the tumor. The levels of TGF-β1, TβRI, p-Smad2, and p-Smad3 were measured in all tissue samples using western blotting. Informed consent was obtained from all patients prior to the collection of samples. The present study was conducted in accordance with the Declaration of Helsinki and approved by the Ethics Committee of Yangpu Hospital, affiliated with Tongji University School of Medicine (clinical study approval No.2019-1369).

### 2.6 Western Blot Analysis

After HeLa and SiHa cells were treated with different concentrations of sildenafil, the old medium was discarded, and the cells were washed three times with PBS solution pre-cooled at 4 ^o^C. 0.2 ml protein extract prepared in advance was added to each well, and the 6-well plate was shaken to spread the lysate evenly over the bottom of the well. After being left to stand on ice for 7 min, all the cell lysates were scraped and transferred to a sterilized and enzyme-free 1. 5-ml Eppendorf tube with a pipette gun head and then placed on ice for 30 min to fully lyse the cells. The speed of the centrifuge was set at 15,000 *×g* for 15 min; after centrifugation, the protein was contained in the supernatant of the upper layer, and cell debris was left at the bottom. The supernatant was transferred to another sterilized 1.5-ml EP centrifuge tube and the total protein was quantified using the bicinchoninic acid (BCA) method. Proteins were resolved on a Sodium dodecyl sulfate (SDS)-denatured polyacrylamide gel (10%) and then transferred onto a nitrocellulose membrane, which was blocked with 5% non-fat milk for 1 h at ^o^C. The membranes were incubated with primary antibodies against snail, vimentin, twist, E-cadherin, N-cadherin, p-Smad2, Smad2, p-Smad3, Smad3, and GAPDH (Sigma-Aldrich; Merck KGaA, St. Louis, MO, USA) overnight at 4 ^o^C. On the next day, the membranes were washed and incubated with a horseradish peroxidase-conjugated secondary antibody (Sigma-Aldrich; Merck KGaA, St. Louis, MO, USA) and visualized using a chemiluminescence ECL western blotting analysis system (BD Biosciences, San Jose, CA, USA). The protein levels were quantified using ImageJ software (v.6.4, National Institutes of Health, Bethesda, MD, USA) after normalization to GAPDH.

### 2.7 Construction of Recombinant Retroviral Vectors and Cell Infection

Similar to the study of Kim *et al.* [24], cDNAs of human TGF-β1, Smad2, and Smad3 were amplified by RT-PCR. The cDNAs were cloned into the pMX retroviral vector (Addgene, Inc., Cambridge, MA, USA) with the recombination method. After the plasmid was confirmed by the DNA sequencing method, the recombinant retrovirus was transduced into HeLa cells. After filtration of the medium from HeLa cell cultures, retroviral particles were concentrated by centrifugation with the Retro X concentrator (cat. #631455, Clonetech, Mountain View, CA, USA) for 1 h at 1,500 ×g at 4 ^o^C. The retroviral particles were then resuspended in 5% FBS/DMEM and stored at -80 ^o^C.

For viral infection, passage 3 HeLa cells were seeded in 6-well plates at a density of 4×10^4^ cells per well. The next day, HeLa cells were transduced with rRV-GFP, rRV-TGF-β1, rRV-Smad2 or rRV-Smad3 at a multiplicity of infection (MOI) of 10 in the presence of 4 μg/ml polybrene (Sigma-Aldrich; Merck KGaA, St. Louis, MO, USA). The efficiency of transduction was evaluated *via* fluorescence microscopy (Leica DMI 3000B; Leica Microsystems GmbH, Wetzlar, Germany).

### 2.8 Xenograft Mice Model and Treatments

A total of 30 BALB/C-nu/nu female nude mice (four weeks, 19-21 g) were kept at the Animal Center of Yangpu Hospital, affiliated with Tongji University School of Medicine. Mice were selected and marked with 1-30 numbers on the back. According to the computer-generated 1-72 random number, the nude mice were divided into six groups with 12 mice in each group. A total of 70-80% of HeLa cells in the logarithmic growth phase were rinsed with PBS twice, 1 ml 0.25% trypsin-0.02% EDTA was added for digestion and counting, the digested cells were collected into the centrifuge tube, centrifuged at 1,200 *×g* for 5 min at room temperature and the supernatant was discarded. 1 ml PBS was added for rinsing, followed by centrifugation at 1,200 *×g* for 5 min at room temperature, the supernatant was discarded, 1 ml normal saline was added for suspension, and the cell concentration was adjusted to 5×10^7^ cells/ml. Each nude mouse was inoculated subcutaneously with 0.2 ml of the cell suspension. As described by de Carvalho *et al.* [25], the mice were repeatedly administered sildenafil (2. 5, 5, 10 and 20 mg/kg) by oral gavage for 7 days. After successful preparation of single-cell suspension, the cage was opened on the super clean platform, the skin on the back of nude mice was disinfected with Iodophor (Sigma) and 0.2 ml of single-cell suspension was injected subcutaneously on the back (n=10 mice per group). Tumor growth was observed 3-5 days after inoculation. The tumor volume was measured in nude mice. To determine the growth rate of the tumor, the time intervals between weighing and measuring tumor volume were determined and recorded in detail. At the 20^th^ day, the nude mice were euthanized by injecting excessive 1% pentobarbital sodium (i.p., 0.05 mL/10 g), and cervical dislocation was performed to confirm death. The tumors were removed with medical scissors and forceps, and a ruler was used as a reference to read the specific scale. The removed tumors were weighed and stored at -80 ^o^C after freezing in liquid nitrogen. The long diameter (L) and the short diameter (W) of the tumor were measured to calculate the tumor volume (V) using the formula V=π/6×L×W×W. The maximum tumor size was <2.0 cm^3^ and the maximum tumor diameter was <1.5 cm. The humane endpoints include the following: rapid bodyweight loss of 15-20% of the original body weight; loss of appetite: mice do not eat at all for 24-36 hours or eat only a small amount of food for 3 days; unable to feed or drink by themselves; an infection that has no good response to medicine treatment and continues to develop into systemic diseases; the tumor weight exceeds 10% of the original body weight, or the average tumor diameter exceeds 20 mm, or the tumor metastasizes or grows rapidly to ulceration, causing infection or necrosis. The animal study was approved by the Ethics Committee of Yangpu Hospital, affiliated to Tongji University School of Medicine (animal study approval No. 2019-1370).

### 2.9 Statistical Analyses

The experimental data are expressed as mean ± standard deviation, and SPSS17.0 software (SPSS, Inc., Chicago, IL, USA) was used for statistical analysis. Comparison of the mean values of multiple groups of data was performed using one-way ANOVA with a completely random design followed by Tukey's post-hoc tests. *p*<0.05 was considered to indicate statistically significant differences.

## 3 RESULTS

### 3.1 Sildenafil Inhibited the Proliferation and Clone-forming Ability of Cells

**Fig. (1):**
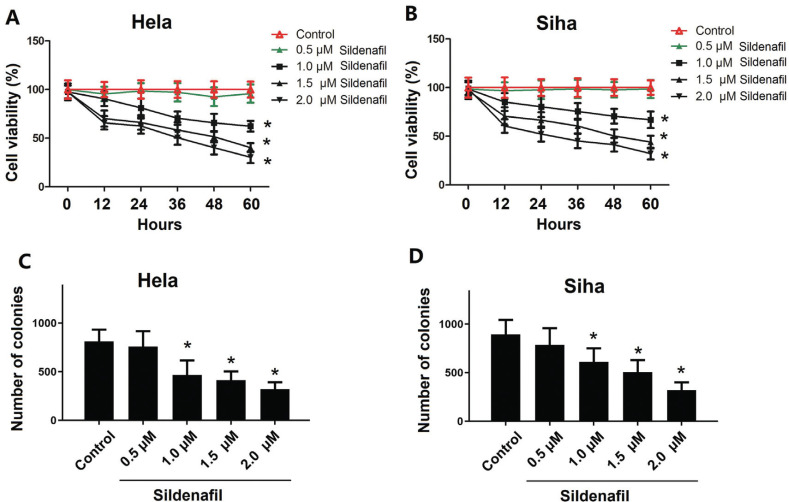
The effects of sildenafil on the proliferation and clonogenicity of HeLa and SiHa cells. The effects of sildenafil on the proliferation of (**A**) HeLa and (**B**) SiHa cells. The effects of sildenafil on the clonogenicity of (**C**) HeLa and (**D**) SiHa cells. The cells in the control group were cultured in normal DMEM with 10% fetal bovine serum. The data are expressed as the mean ± standard error (n=10). *: *p*<0.05 compared with the control group.

High concentrations of sildenafil (1.0-2.0 μM) significantly inhibited the proliferation of HeLa and SiHa cells (*p*<0.05, Fig. **1A**, **B**). The viability of both HeLa and SiHa cells exhibited a significant incubation duration-dependent decrease. As shown in Fig. (**1C**, **1D**), the number of HeLa and SiHa colonies was significantly decreased by sildenafil (1.0-2.0 μM), whereas low concentrations of sildenafil (0.5 μM) did not significantly inhibit the proliferation or clone-forming ability of the cells.

### 3.2 Sildenafil Decreased the Migration and Invasion and Invasion of HeLa and SiHa Cells

**Fig. (2):**
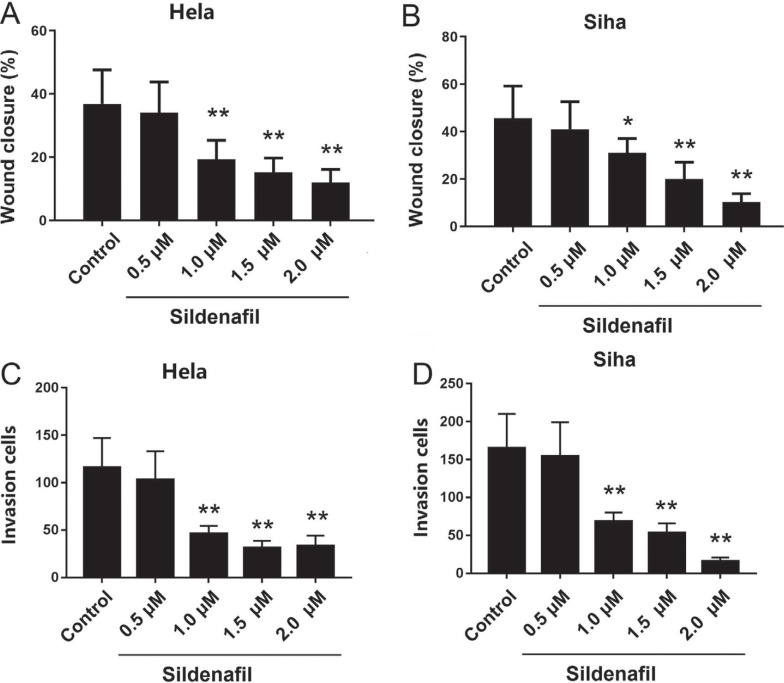
The effects of sildenafil on the migratory and invasive activities of HeLa and SiHa cells. The effect of sildenafil on the wound closure rate of (**A**) HeLa and (**B**) SiHa cells. The effects of sildenafil on the invasive activities of (**C**) HeLa and (**D**) SiHa cells. The cells in the control group were cultured in normal DMEM without fetal bovine serum. The data are expressed as the mean ± standard error (n=10). *: *p*<0.05 compared with control cells. **: *p*<0.01 compared with the control group. Scar bar=20 μM.

The results of the wound healing (Fig. **2A**, **B**, Supplementary Fig. (1A) and Transwell assays (Fig. **2C**, **D**, Supplementary Fig. **1B**) are shown in Fig. (**2**) and Supplementary Fig. (1). It was shown that 0.5 μM sildenafil did not significantly change the wound closure rate compared to control, but 1.0-2.0 μM sildenafil significantly decreased the wound closure rates in HeLa and SiHa cells (*p*<0.05). The number of invading cells was not significantly changed by 0.5 μM sildenafil but was significantly decreased by sildenafil (1.0-2.0 μM) compared with the control (*p*<0.05, Fig. **2C**-**D**, **F**).

### 3.3 Expression of TGF-β1, TβRI, p-Smad2 and p-Smad3 in Cervical Cancer Tissues and Cervical Cancer Cells

**Fig. (3):**
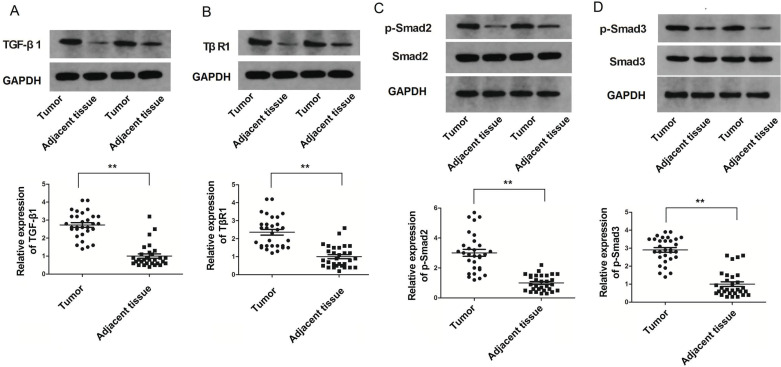
The expression levels of TGF-β1, TβRI, p-SMAD2 and p-SMAD3 in cervical cancer tissues. (**A**) The expression levels of TGF-β1 in cervical cancer and normal cervical tissues measured by Western blot. (**B**) The expression levels of TβRI in cervical cancer and normal cervical tissues measured by Western blot. (**C**) The expression levels of p-SMAD2 and SMAD2 in cervical cancer and normal cervical tissues measured by Western blot. (**D**) The expression levels of p-SMAD3 and SMAD3 in cervical cancer and normal cervical tissues measured by Western blot. The data are expressed as the mean ± standard error (n=10). **: *p*<0.01 compared with control cells. TGF-β1, transforming growth factor-β1; TβRI, transforming growth factor β type I receptor; p-, phosphorylated. **: *p*<0.01 compared with the control group.

**Fig. (4):**
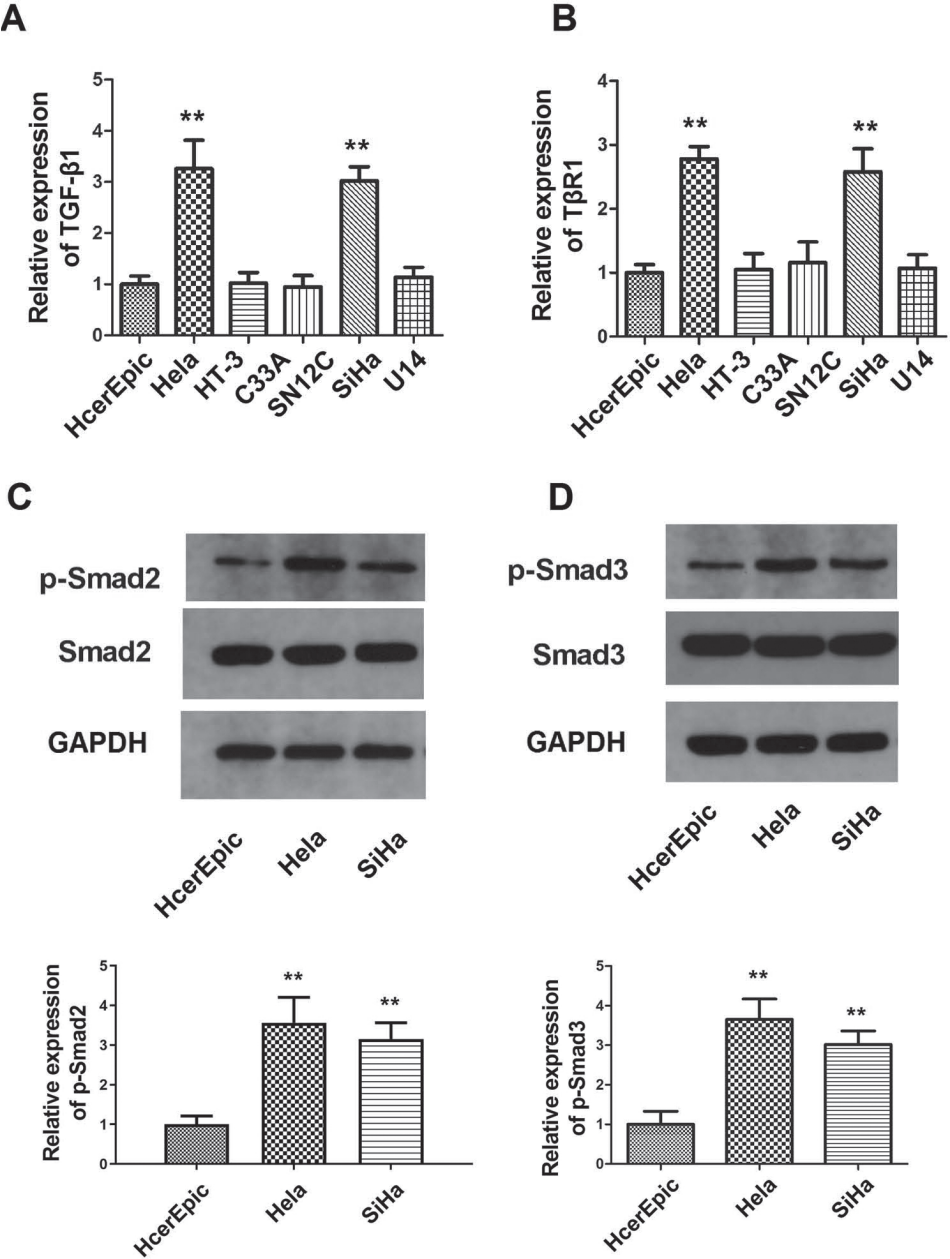
The expression levels of TGF-β1, TβRI, p-SMAD2 and p-SMAD3 in cervical cancer cells. (**A**) The mRNA expression levels of TGF-β1 in cervical cancer cells measured by qPCR method. (**B**) The mRNA expression levels of TβRI in cervical cancer cells measured by qPCR method. (**C**) The expression levels of p-SMAD2 and SMAD2 in HeLa and SiHa cells measured by Western blot. (**D**) The expression levels of p-SMAD3 and SMAD3 in HeLa and SiHa cells measured by Western blot. The data are expressed as the mean ± standard error (n=10). **: *p*<0.01 compared with control cells. TGF-β1, transforming growth factor-β1; TβRI, transforming growth factor β type I receptor; p-, phosphorylated. **: *p*<0.01 compared with the control group.

The expression of TGF-β1, TβRI, p-Smad2 and p-Smad3 were analyzed in a total of 30 cervical cancer and normal tissue samples. As demonstrated in Fig. (**3A**-**D**), the expression of TGF-β1, TβRI, p-Smad2 and p-Smad3 was significantly enhanced in cervical cancer samples (*p*<0.05; Fig. **3A**-**D**). To confirm whether the TGF-β1/Smad2/3 pathway was activated in cervical cancer cells, we examined the expression of TGF-β1 and TβRI in cervical cancer cell lines (HeLa, HT-3, C33A, SiHa and U14) and HCerEpiC cells. The results revealed that the expression of TGF-β1 and TβRI was significantly increased in HeLa and SiHa cells (*p*<0.05; Fig. **4**). Next, the phosphorylation levels of Smad2/3 (p-Smad2 and p-Smad3) were measured in HeLa and SiHa cells. The results revealed that, compared with HCerEpiC cells, the phosphorylation levels of Smad2/3 (p-Smad2 and p-Smad3) in HeLa and SiHa cells were significantly increased (*p*<0.05); (Fig. **3G**-**H**).

### 3.4 Sildenafil Inhibited TGF-β1-Induced EMT and Smad2/3 Phosphorylation in HeLa Cells

**Fig. (5):**
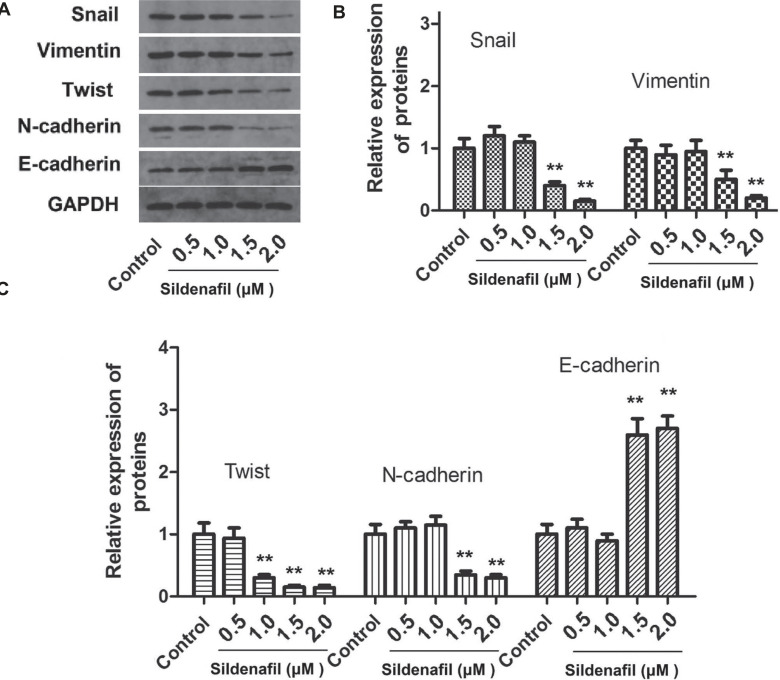
The effects of sildenafil on TGF-β1-induced EMT in HeLa cells. (**A**) Representative images of the western blots corresponding to the expression of the EMT-associated marker proteins (Snail, vimentin, Twist, E-cadherin and N-cadherin). (**B**) The results of the expression analysis of Snail, vimentin proteins in HeLa cells. (**C**) The results of the expression analysis of Twist, E-cadherin and N-cadherin proteins in HeLa cells. The cells in the control group were cultured in normal DMEM with 10% fetal bovine serum. The data are expressed as mean ± standard error (n=10). *: *p*<0.05 compared with control; **: *p*<0.01 compared with the control group. EMT, epithelial-mesenchymal transition; Snail, snail family transcriptional repressor 1; p-, phosphorylated; Twist, twist family bHLH transcription factor 1.

After HeLa cells were treated with sildenafil and TGF-β1, the levels of the EMT marker proteins (Snail, vimentin, Twist, E-cadherin and N-cadherin) and the expression of TGF-β1, TβRI, p-Smad2, Smad2, p-Smad3, Smad3 in HeLa cells were measured using western blotting. After HeLa cells were treated with sildenafil, the levels of the EMT marker proteins (Snail, vimentin, Twist, E-cadherin and N-cadherin) in HeLa cells were measured using western blotting. As shown in Fig. (**5**), high concentrations of sildenafil (1.0-2.0 μM) significantly decreased the expression of snail, vimentin, twist, and N-cadherin and increased the expression of E-cadherin in HeLa cells (*p*<0.05, Fig. **5**), but not significantly changed by 0.5 μM sildenafil (*p*>0.05 compared to Control). As shown in Fig. (**6**), the expression of TGF-β1 and TβRI was significantly decreased by sildenafil (1.0-2.0 μM) but not significantly changed by 0.5 μM sildenafil (*p*>0.05 compared to control). As shown in Fig. (**6**), the expression of p-Smad2 and p-Smad3 was significantly decreased by sildenafil (1.0-2.0 μM) but not significantly changed by 0.5 μM sildenafil (*p*>0.05 compared to control).

### 3.5 Overexpression of TGF-β1, Smad2 and Smad3 Reversed the Effect of Sildenafil on EMT in HeLa Cells

**Fig. (7):**
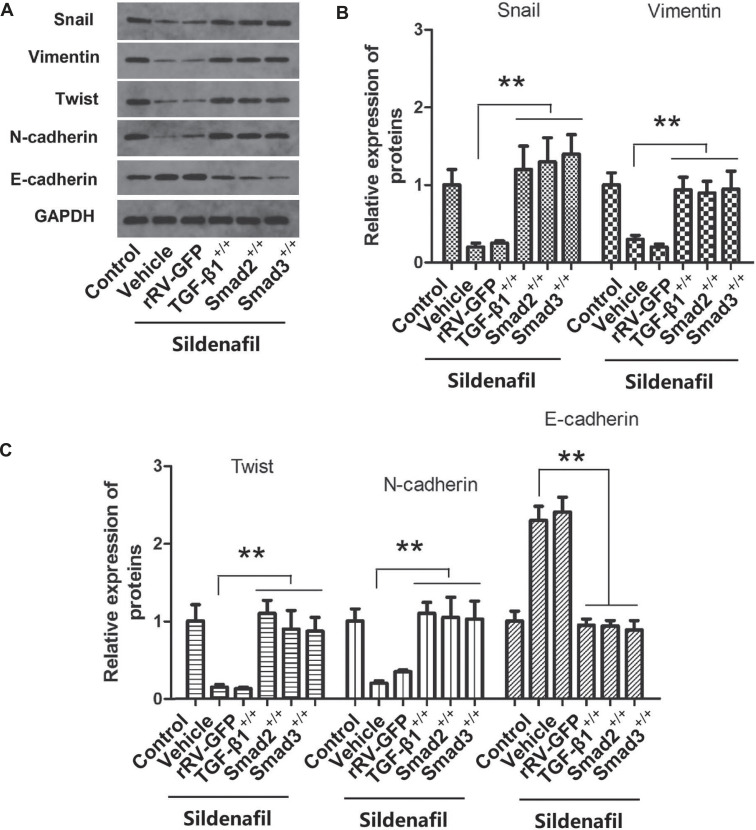
The effects of TGF-β1, SMAD2 and SMAD3 overexpression and sildenafil treatment on EMT in HeLa cells. (**A**) Representative images corresponding to the western blot experiments. (**B-C**) The results of the expression analysis of the EMT-associated marker proteins (Snail, vimentin, Twist, E-cadherin, N-cadherin) in HeLa cells. The cells in the control group were cultured in normal DMEM with 10% fetal bovine serum. The data are expressed as mean ± standard error (n=10). **: *p*<0.01 compared with the control group. TGF-β1, transforming growth factor-β1; EMT, epithelial-mesenchymal transition; Snail, snail family transcriptional repressor 1; Twist, twist family bHLH transcription factor 1.

After HeLa cells were treated with a plasmid containing TGF-β1, Smad2, and Smad3, they were treated with sildenafil and TGF-β1, and then the expression of EMT-related marker proteins (Snail, vimentin, Twist, E-cadherin and N-cadherin) in HeLa cells was measured using western blotting. As shown in Fig. (**7**), compared with Vehicle, rRV-GFP did not significantly change the expression levels of EMT marker proteins. Overexpression of TGF-β1, Smad2 and Smad3 increased the expression of snail, vimentin, twist, and N-cadherin in the cells and inhibited the expression of E-cadherin (*p*<0.05, Fig. **7**).

### 3.6 Overexpression of TGF-β1, Smad2 and Smad3 Reversed the Effect of Sildenafil on the Viability, Colony Formation, Migration, and Invasion Ability of HeLa Cells

**Fig. (8):**
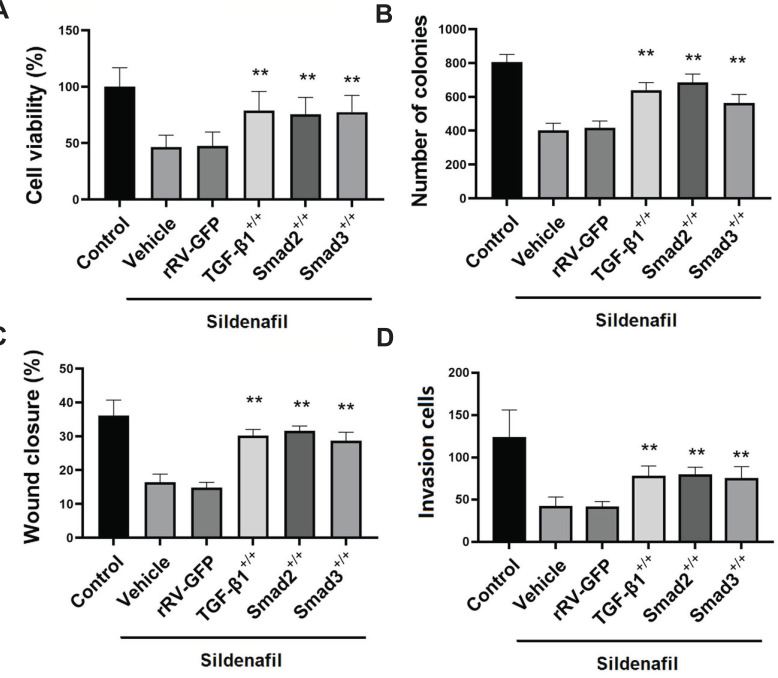
Overexpression of TGF-β1, SMAD2 and SMAD3 reverses the effects of sildenafil on viability, clonogenicity and migratory and invasive activities of HeLa cells. (**A**) Estimation of the viability of HeLa cells. (**B**) Estimation of the number of HeLa cell colonies. (**C**) Determination of the wound healing rate of SiHa cells. (**D**) Determination of the invasive activity of HeLa cells. The cells in the control groups were cultured in normal DMEM with 10% FBS for determination of cell viability and clonogenicity. The cells in the control groups were cultured in normal DMEM without FBS for the determination of migratory and invasive activities. The data are expressed as mean ± standard error (n=10). **: *p*<0.01 compared with control cells. TGF-β1, transforming growth factor-β1, FBS, fetal bovine serum.

After HeLa cells were treated with a plasmid containing TGF-β1, Smad2, and Smad3, they were treated with sildenafil, and then the viability, colony formation, migration, and invasion abilities of HeLa cells were measured. As shown in Fig. (**8**), compared with Vehicle, rRV-GFP did not significantly change the viability, colony formation, migration (wound closure rate) or invasion (invasion cell count) abilities of HeLa cells, whereas overexpression of TGF-β1, Smad2, and Smad3 significantly increased the viability, colony formation, migration (wound closure rate), and invasion (invasion cell count) abilities of HeLa cells (*p*<0.05 compared to control, Fig. **8**).

### 3.7 Sildenafil Increased Mouse Survival Rates and Suppressed Xenograft Tumor Growth

**Fig. (9):**
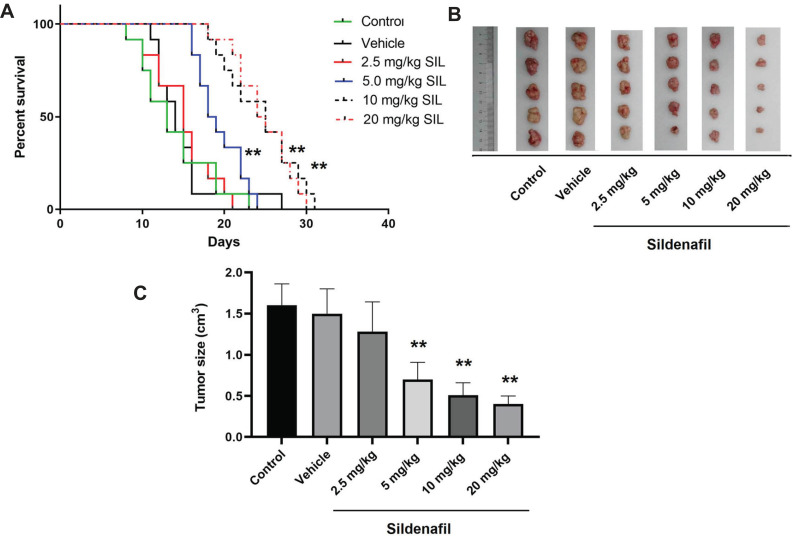
Effects of sildenafil on mouse survival rate and xenograft tumor growth. Nude mice were subcutaneously injected with HeLa cells to establish xenograft tumors. The mice were subsequently treated with sildenafil. The survival rates (**A**) and tumor size (**B, C**) were measured. The mice in the control group received no treatment. The data are expressed as mean ± standard error (n=12 per group). **, *p*<0.01 compared with the control group.

Changes in body weight, survival rate, tumor size and tumor inhibitory rate were evaluated in xenograft mice treated with different concentrations of sildenafil. The survival rate in the sildenafil groups was significantly higher compared with the control (*p*<0.05; Fig. **9A**). As shown in Fig. (**9B**), the tumor sizes in the sildenafil groups were significantly lower compared with the control. As shown in Fig. (**9C**), the tumor inhibitory rate of the sildenafil groups was significantly higher compared with the control.

## 4 DISCUSSION

In recent years, studies have reported that sildenafil can inhibit the growth of multiple cancers. The study of Islam *et al.* revealed that sildenafil suppresses polyp formation and inflammation in mice treated with azoxymethane/dextran sulfate sodium, indicating that PDE5 as a target highlights the potential therapeutic value of PDE5 inhibitors for the prevention of colon cancer [26]. Sildenafil can activate CD95, thereby activating the JNK signaling pathway, leading to apoptosis of tumor cells [27]. Another study showed that sildenafil induced cell death of various cancer cells (MDA-MB-231, SW480, MIA PaCa-2, Panc1, BxPC3 and A549 cells) by changing the expression of HSP90 and degradation of PKD2 [28]. In addition, sildenafil can improve the antitumor effect of doxorubicin in prostate cancer, as well as the antitumor effect of doxorubicin, cisplatin, and mitomycin in the bladder and pancreatic cancer [29]. Furthermore, it was also reported that sildenafil decreased the levels of TGF-β1 in a model of renovascular hypertension [30]. Sildenafil treatment also counteracted the increased expression of TGF-β1 and Bax in the kidneys of DOCA-salt hypertensive rats [31]. In the present study, sildenafil was shown to inhibit the proliferation of HeLa and SiHa cervical cancer cells. Sildenafil also significantly inhibited the migration and invasion of HeLa and SiHa cells. These preliminary results suggested that sildenafil may inhibit the migration and invasion of cervical cancer.

The TGF-β1 pathway is a key to a number of cellular processes, including cell proliferation, migration, and apoptosis [32, 33]. It was previously demonstrated that TGF-β1 promotes breast cancer invasion, metastasis, and epithelial-mesenchymal transition by autophagy [34]. In most tumors, the TGF-β1 signal transduction pathway is not affected, but the tumor cells are resistant to the anti-proliferative effect of TGF-β1. As a result, TGF-β1 does not inhibit proliferation but serves as a carcinogen [35]. In addition, TGF-β1 can induce and block the proliferation and differentiation of immune cells. Therefore, in tumors that are resistant to the anti-proliferative effect of TGF-β1, TGF-β1 can promote tumor formation by blocking the antitumor immune response [36, 37]. There are three types of TGF-β1 receptors, namely TβRI, TβRII, and TβRIII [38]. TβRI is composed of a signal peptide, hydrophilic region, and transmembrane region. It is also a part of the serine/threonine kinase family of receptors, which regulate the signaling pathway transmission [39]. To investigate the role of TGF-β1 and TβRI in the development of cervical cancer, the levels of TGF-β1, TβRI, p-Smad2, and p-Smad3 were measured in cervical cancer and adjacent tissues and were found to be significantly higher in cervical cancer issues. In addition, the protein levels of TGF-β1 and TβRI and the phosphorylation levels of Smad2/3 (p-Smad2 and p-Smad3) were significantly increased in HeLa and SiHa cells, indicating that the TGF-β1/Smad2/3 pathway was activated in cervical cancer.

TGF-β1 binds to the TβRII receptor on the cell membrane. After binding, TβRII is activated and TβRI is recruited to form a trimer complex of TGF-β-TβRII-TβRI, which phosphorylates TβRI and further transmits the signal to Smads [32]. The function of the Smad proteins is to transfer the signal in the pathway from the cell membrane to the nucleus, and three classes of Smads, including receptor-regulated Smads (R-Smads, Smad1–Smad3, Smad5 and Smad8), common mediator Smads (Co-Smads, Smad 4), and inhibitory Smads (I-Smads, Smad 6 and Smad 7) have been identified in biological system [40]. TGF-β binds to TGF-βRII and recruits TGF-βRI in the plasma membrane. Then, Smad2/3 was phosphorylated and bound to Smad4 to transmit signals to the nucleus [41]. Inactivation of Smad2 and Smad4 is often found in some tumors, including missense, nonsense codon, frame shift mutations and deletions. The majority of the missense mutations occurred in the Smad MH2 domain and inhibited the formation of the Smad2/3-Smad4 complex [42]. The results of the present study demonstrated that the expression of p-Smad2 and p-Smad3 was significantly decreased by sildenafil (1.0, 1.5 and 2.0 μM), indicating that sildenafil inhibits the TGF-β1/Smad2/3 pathway in HeLa cells.

Previous studies have confirmed that the EMT pathway is also regulated by the TGF-β/Smad signaling pathway [43, 44]. To examine the role of EMT in the effect of sildenafil on cervical cancer, HeLa cells were treated with different concentrations of sildenafil and TGF-β1, and the expression of the EMT marker proteins (Snail, vimentin, Twist, E-cadherin and N-cadherin) was then measured. As shown in (Fig. **4**), sildenafil suppressed TGF-β1-induced EMT in HeLa cells. The role of Smad2/3 in EMT has been previously investigated. Kim *et al.* found that, in breast cancer cells (MCF-7), EGF stimulated the expression of snail, vimentin and fibronectin, but decreased the expression of E-cadherin [45]. Kim *et al.* knocked down the expression of Smad2/3 and then observed that the expressions of snail, vimentin and fibronectin were reduced [45], verifying that the activation of Smad2/3 can increase the expression of EMT key marker proteins and inhibit the expression of E-cadherin. Inhibition of E-cadherin stimulated EMT and the migration and invasion of breast cancer cells [44]. Another study confirmed that overexpression of GRP78 promotes the secretion and expression of TGF-β1 in human colon cancer cell line (DLD1) DLD1 cells, activates the downstream Smad2/3 signal transduction pathway, and promotes cell matrix adhesion and EMT [43]. Furthermore, Zhang *et al.* found that, in Hep3B cells, when mitochondria are damaged, EMT may be induced through the TGF-β/Smad/snail signaling pathway [43]. In the present study, the results of western blotting also confirmed that the expression of p-Smad2 and p-Smad3 in the Smad pathway decreased significantly after EMT was induced by TGF-β1, while the total expression levels of Smad2 and Smad3 did not change. These results indicate that sildenafil suppresses the phosphorylation of Smad2/3 in the Smad pathway and regulates the EMT process of cervical cancer cells. To verify the role of the TGF-β1/Smad2/3 pathway in the antitumor effects of sildenafil, we overexpressed TGF-β1, Smad2 and Smad3 in HeLa cells treated with sildenafil, then measured the expression of EMT marker proteins, the viability, colony formation, migration and invasion abilities. These results indicated that overexpression of TGF-β1, Smad2 and Smad3 reversed the effect of sildenafil on EMT, viability, colony formation, migration and invasion abilities of HeLa cells. Therefore, the antitumor effect of sildenafil is mediated through TGF-β1, Smad2 and Smad3.

Finally, to confirm the antitumor effect of sildenafil *in vivo*, HeLa cells were used to establish a nude mouse xenograft model treated with sildenafil. Sildenafil significantly increased the tumor inhibitory rate and the survival rate of mice but decreased the tumor size. These results suggested that sildenafil can also inhibit cervical cancer progression *in vivo*. Together with the results of the *in vitro* experiments, these findings indicated that sildenafil may inhibit the proliferation and invasion/migration ability of human cervical cancer cells, as well as inhibit the EMT process in HeLa cells by regulating the TGF-β1/Smad2/3 pathway. This suggests that sildenafil may represent a novel promising agent for the treatment of cervical cancer.

## CONCLUSION

Furthermore, this newly identified mechanism may provide further insight into the development of cervical cancer. Overall, this study revealed the anti-cancer potential of sildenafil and the application prospect of sildenafil in the field of cervical cancer. Based on the results in the present study, some preclinical studies can be designed to further confirm the effect and safety of sildenafil in the treatment of cervical cancer. However, there are several limitations in the study that need to be noted. Firstly, the specific mechanism that sildenafil regulates the TGF-β1/Smad2/3 pathway needs to be investigated in the future. Secondly, EMT may not be the only mechanism that sildenafil inhibits cervical cancer. Thirdly, it is necessary to explore the dosage and duration of sildenafil treatment in future clinical research.

## Figures and Tables

**Fig. (6):**
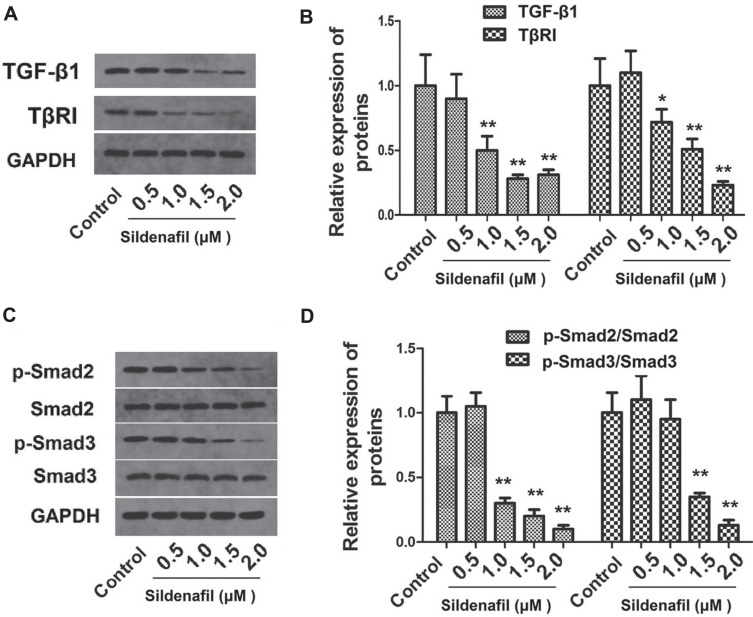
The effects of sildenafil on the TGF-β1/SMAD2/3 signaling pathway in HeLa cells. (**A**) Representative images of western blots corresponding to TGF-β1 and TβRI expression. (**B**) Semi-quantification of the expression levels of TGF-β1 and TβRI protein in HeLa cells. (**C**) Representative images of the western blots for p-SMAD2, SMAD2, p-SMAD3 and SMAD3. (**D**) Semi-quantification of SMAD2/3 phosphorylation levels in HeLa cells. The cells in the control group were cultured in normal DMEM with 10% fetal bovine serum. The data are expressed as mean ± standard error (n=10). *: *p*<0.05 compared with control; **: *p*<0.01 compared with the control group. TGF-β1, transforming growth factor-β1; TβRI, transforming growth factor β type I receptor; p-, phosphorylated.

## Data Availability

The data used to support the findings of this study are available from the corresponding author (Dr. Xiao-Ping Ke) upon request.

## LIST OF ABBREVIATIONS

EMT: Epithelial-to-Mesenchymal Transition
TβRI: Transforming Growth Factor-β Type I Receptor
PDE5: Phosphodiesterase 5
