# Supplementary material for: Sildenafil Inhibits the Growth and Epithelial-to-mesenchymal Transition of Cervical Cancer via the TGF-β1/Smad2/3 Pathway
Source: Curr Cancer Drug Targets. 2023 Jan 1;23(2):145–58. doi: 10.2174/1568009622666220816114543 (PMC10173468; doi:10.2174/1568009622666220816114543)
Supplement: Supplementary file 1 [file CCDT-23-145_SD1.pdf]

Supplementary Materials

Sildenafil Inhibits the Growth and Epithelial-to-Mesenchymal Transition of Cervical Cancer *via* the TGF-β1/Smad2/3 Pathway

Ping Liu[1,#], Jing-Jing Pei[2,#], Li Li[1], Jing-Wei Li[1] and Xiao-Ping Ke[1,*]

[1]*Department of Obstetrics and Gynecology, Yangpu Hospital, School of Medicine, Tongji University, Shanghai, 200090, China;* [2]*Child Healthcare Department, The Affiliated Wuxi Matemity and Child Health Care Hospital of Nanjing Medical University, Wuxi 214001, China*

SUPPLEMENTARY FIG. S1

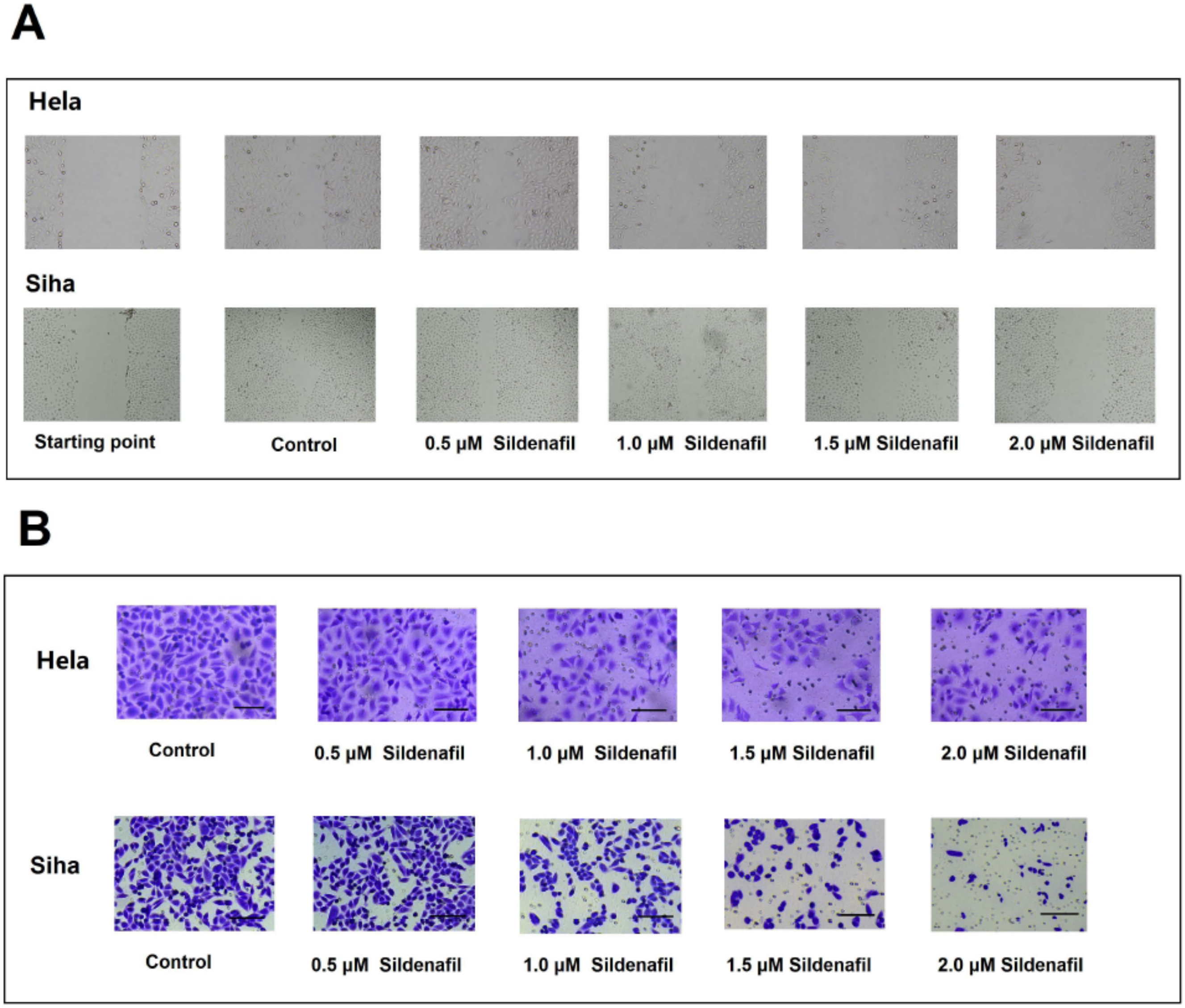

Supplementary Fig. (S1). The representative images of wound healing and Transwell assay. **A**, representative images of wound healing assay; **B**, representative images of Transwell assay.

 © 2023 Bentham Science Publishers
